# Unveiling FAD^3T^: A Groundbreaking Transgenic Mouse Model for Accelerating Alzheimer's Disease Research

**DOI:** 10.1002/alz70855_102567

**Published:** 2025-12-23

**Authors:** Ziying Lai, Bingzhou Han, Tao Wang, Xiang Gao, Rui Feng

**Affiliations:** ^1^ GemPharmatech Co., Ltd., Nanjing, Jiangsu, China; ^2^ GemPharmatech Co., Ltd., Guangdong, Guangdong, China; ^3^ Nanjing University, Nanjing, Jiangsu, China; ^4^ Jiangsu Industrial Technology Research Institute, Nanjing, Jiangsu, China

## Abstract

**Background:**

Alzheimer's disease (AD) poses a significant challenge in contemporary healthcare, with traditional mouse models often exhibiting prolonged disease progression periods that impede efficient pharmaceutical drug discovery and development.

**Methods:**

To address this challenge, we developed the FAD^3T^ transgenic mouse model, incorporating three mutations (hAPP, hPSEN1, hMAPT) that closely replicate key genetic factors implicated in AD pathogenesis.

**Results:**

The FAD^3T^ model exhibits phenotypic parallels to human AD patients, with early onset of pathogenic markers. Notably, diagnostic blood markers such as *p*‐Tau181, *p*‐Tau217, Aβ42/40, and neurofilament light chain (NfL) are detectable in the plasma of 1‐month‐old FAD^3T^ mice. Elevated levels of phosphorylated Tau (Thr181) are observed in the brain at 1 month of age. Subsequent observations include Aβ deposition and elevated levels of Phospho‐Tau (Thr217, Thr231) at 2 months, followed by elevated levels of Phospho‐Tau (Ser396, Ser202, Thr205) at 3 months, with all markers progressively intensifying with age. Importantly, cognitive deficits, including spatial learning and memory impairments, manifest at earlier stages in these mice.

**Conclusion:**

Our findings highlight the exceptional fidelity of the FAD^3T^ model in recapitulating the complex etiology of AD. Compared to traditional AD mouse models, FAD^3T^ exhibits accelerated disease onset and progression, providing a rapid platform for expediting drug testing cycles, particularly for therapies targeting disease progression. This innovative mouse model represents a transformative tool for enhancing our understanding of AD pathophysiology and facilitating the expedited development of therapeutic strategies.

